# A Study on Seizure Detection of EEG Signals Represented in 2D

**DOI:** 10.3390/s21155145

**Published:** 2021-07-29

**Authors:** Zhiwen Xiong, Huibin Wang, Lili Zhang, Tanghuai Fan, Jie Shen, Yue Zhao, Yang Liu, Qi Wu

**Affiliations:** 1 College of Computer and Information Engineering, Hohai University, Nanjing 211100, China; xzw@hhu.edu.cn (Z.X.); lilzhang@hhu.edu.cn (L.Z.); shenjie_2003045@hhu.edu.cn (J.S.); 2 School of Information Engineering, Nanchang Institute of Technology, Nanchang 330029, China; fantanghuai@nit.edu.cn; 3 School of Computer Science and Technology, Hangzhou Dianzi University, Hangzhou 310018, China; zhaoyue@hdu.edu.cn (Y.Z.); liuyangcn@hdu.edu.cn (Y.L.); wuqi@hdu.edu.cn (Q.W.)

**Keywords:** EfficientNet neural network, seizure detection, epilepsy, EEG feature representation

## Abstract

A seizure is a neurological disorder caused by abnormal neuronal discharges in the brain, which severely reduces the quality of life of patients and often endangers their lives. Automatic seizure detection is an important research area in the treatment of seizure and is a prerequisite for seizure intervention. Deep learning has been widely used for automatic detection of seizures, and many related research works decomposed the electroencephalogram (EEG) raw signal with a time window to obtain EEG signal slices, then performed feature extraction on the slices, and represented the obtained features as input data for neural networks. There are various methods for EEG signal decomposition, feature extraction, and representation, and most of the studies have been based on fixed hardware resources for the design of the scheme, which reduces the adaptability of the scheme in different application scenarios and makes it difficult to optimize the algorithms in the scheme. To address the above issues, this paper proposes a deep learning-based model for seizure detection, mainly characterized by the two-dimensional representation of EEG features and the scalability of neural networks. The model modularizes the main steps of seizure detection and improves the adaptability of the model to different hardware resource constraints, in order to increase the convenience of the algorithm optimization or the replacement of each module. The proposed model consists of five parts, and the model was tested using two epilepsy datasets separately. The experimental results showed that the proposed model has strong generality and good classification accuracy for seizure detection.

## 1. Introduction

Between 1% and 2% of the world’s population endures seizures, which are temporary events caused by abnormal neuronal activity in the brain. Seizures are unpredictable for patients in their daily lives and are a potential cause of their disability or even death. Seizures are emotionally stressful for patients and their families, and treatment is costly and expensive. There are many methods used for the treatment and rehabilitation of seizure, some of which are daily medications provided to patients to reduce the number of seizures, such as the use of the drug Epidiolex. There are also treatments that reduce the extent of seizures, such as electrical stimulation, including vagus nerve stimulation and responsive neurostimulation. To apply therapies during seizures, seizure detection is a prerequisite. Therefore, many researchers have focused on the automatic detection of seizures [1], and many electroencephalogram(EEG)-based classification schemes have been generated.

EEG is an important means of recording neuronal activity in the brain and contains information that reflects the overall activity of the cerebral cortex. Because the EEG during a seizure is different from when the brain is normal, the EEG can be used to detect seizures. This is important for carrying out automatic closed-loop treatment of seizure and is one of the key technologies for developing artificial intelligence systems and tools for the treatment of seizure. In addition, seizure detection can also be used for the assessment of the effect of patient treatment.

Deep learning is a widely followed machine learning paradigm that works in a multilevel combination and obtains high-level features from the input data. Deep learning does not require the manual design of the corresponding feature extractor for each classification problem. The structure of a deep neural network always contains more than two implicit layers, and the weights of each layer are adjusted according to the input data during training. The trained network can produce corresponding classification results for different input data. Deep learning techniques are now widely used for EEG signal classification, including seizure detection. Most of the related studies first use the EEG data to generate the input data for the network and then train the network to acquire the ability to classify the EEG. Because of the black-box nature of neural networks, it is always difficult for a network to reduce the dimensionality of the input data, so it is not always appropriate to use the raw EEG data as the input data. Different kinds of input data can affect the classification performance of neural networks. Therefore, many studies decompose the original EEG to obtain many slices of the EEG signal and then use each slice to extract features in the time domain, frequency domain, time–frequency domain, etc. The representation of the features are the input data of the neural network. The input data can be one-dimensional, two-dimensional, or high-dimensional. The input data of different dimensions have different processing complexities. We want the input data to retain the important information in the slices and have a lower dimensionality. After training, the trained neural network will obtain new deep features, and the classifier calculates the classification results based on the depth of the features. The obtained results can be used as the classification results of slices and for further processing to obtain the classification results of EEG signals.

Although there are many studies that have used deep learning to classify EEG signals, most of them have designed their schemes based on the hardware resources they have, which makes it impossible to scale the width, depth, and resolution of the neural network when these schemes are deployed on different hardware platforms. In addition, because of the fixed structure of the neural network, the form of the input data representation is closely related to the structure of the neural network, which likewise reduces the possibility of reusing a network’s structure or the input data’s representation algorithm in future research. If the coupling among different technical parts in a scheme is stronger, it is more difficult to replace different parts in the future using new algorithms, which also leads to the difficulty of optimizing this scheme. It is the motivation of this paper to make the proposed scheme better adapted to different hardware resource constraints and to facilitate the introduction of new algorithms for scheme optimization.

**Figure 1:**
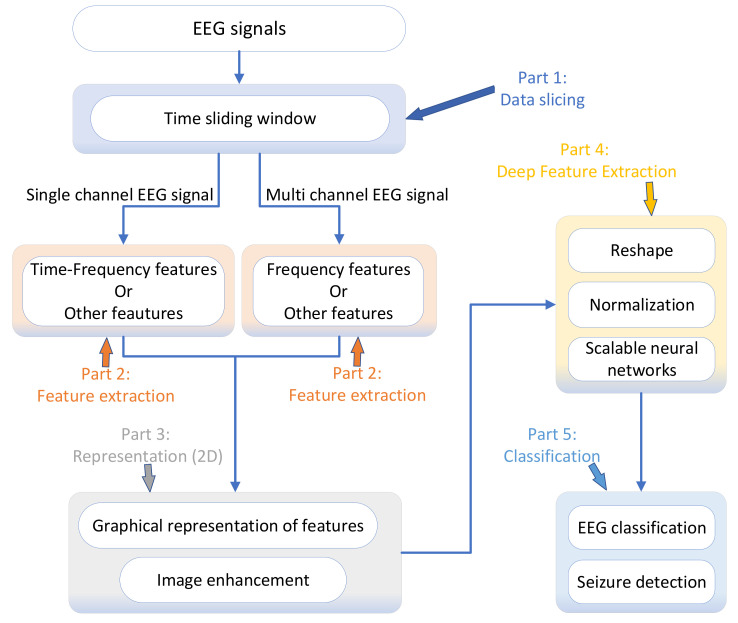
The main parts of the proposed model.

In this paper, we propose a deep learning-based model with two core features: the two-dimensional representation of EEG features and the scalability of the neural network. The EfficientNet neural network [2] was introduced into the model, and this was done for two purposes. One purpose was to improve the classification performance of the model by using the network’s transfer learning capability, and the other purpose was to enhance the model’s ability to adapt to different hardware resource constraints and different kinds of input data by using the ability that the depth, width, and resolution of the network can be changed dynamically to extract more information to improve the classification performance of the model. The model is mainly divided into five parts. They are EEG decomposition, feature extraction, 2D representation, depth feature extraction, and classification, as shown in Figure 1. The first part is EEG decomposition, which uses time windows to decompose EEG signals into segments, called slices. In this paper, we explored the effect of different lengths of time windows on the detection results in epilepsy detection. The second part is feature extraction. Currently, the main EEG signals can be divided into single-channel and multichannel based on the number of channels. For seizure detection, we used different feature extraction methods for single-channel and multiple channel to extract the features in the slices. The third part is the two-dimensional representation of the obtained features, and the representation results were used as the input data of the neural network. In this paper, the result of the 2D representation of the features was a picture, which is a 2D matrix with three channels. The fourth part is the deep feature extraction, which uses the neural network to perform deep feature extraction on representations. The model scales the neural network according to the hardware resource constraints and the characteristics of input data. These scaling factors include the depth, width, and resolution of the network. The fifth part is the classification, which uses the depth features to obtain the classification results of the slices, and this result can be used as a judgment about whether a seizure has been detected or not. The model was tested on two datasets to determine its generality and effectiveness.

The main contributions of this paper are as follows:

1. A deep learning-based seizure detection model is proposed with the 2D representation of a single-channel or multichannel EEG features. Modularizing the model facilitates the algorithm optimization or algorithm replacement for different parts of the model;
2. The EfficientNet network architecture was incorporated into the proposed model, thus allowing the model to adjust the width, depth, and resolution of the network according to different hardware resource limitations and types of input data;
3. Experiments were conducted on two publicly available epilepsy datasets. The settings of some important parameters are explored in the paper, and the effect of transfer learning on the model performance is briefly discussed.

The remainder of this paper is organized as follows. Section 2 presents the main technical background of seizure detection involved in the proposed model. The details of the proposed model are given in Section 3. Section 4 describes the experimental studies, and the results are analyzed in Section 5. Finally, conclusions and future work are given in the last section.

## 2. Related Works

With the development of biosensor technology [3,4], EEG is increasingly used for seizure detection. EEG is a recording of the electrical signals produced by the brain using electrodes to reflect the activity of neurons in the brain. Depending on the placement of the electrodes, EEG can be classified as intracranial EEG and scalp EEG. The former is an EEG signal collected with electrodes inserted onto the brain and placed under the skull, while the latter is an EEG signal collected with electrodes placed immediately on the scalp. It is important to note that some studies have further differentiated the types of scalp EEG based on the location of the electrodes or the complexity of how the device is worn, for example scalp EEG or behind-the-ear EEG [5].

The EEG is different during seizure and nonseizure periods, so an automated algorithm can be used to analyze the EEG signal and determine whether a seizure is present or not. Starting from the early 20th century, some researchers began to study seizures based on EEG. With the rapid development of computer technology, many techniques for automatic seizure identification have emerged. Time domain features, frequency domain features, time–frequency domain features, or nonlinear dynamic features are extracted, and then, these features are used for seizure detection.

Time domain analysis was applied to the analysis of EEG signals early on, mainly using waveform features and rhythm features for seizure detection. In 1982, Gotman extracted the time domain features of the EEG signal: amplitude, peak, slope, and so on, and compared these features with a preset threshold to ultimately determine whether a seizure was occurring [6]. In [7], the authors used a moving window to dynamically analyze the brain based on the analysis of the positive zero-crossing intervals in the scalp EEG. The scheme [8] proposed by Bedeeuzzaman et al. was based on a statistical feature set, performing mean absolute deviation and interquartile range operations, then using a linear classifier to achieve the classification of the seizures. In the paper [9], a fusion method of variational mode decomposition and autoregression was used to extract features, and then, a random forest classifier was used to classify seizures. The authors of [10] combined empirical mode decomposition and the autoregressive model to construct an EEG-based classification model. Firstly, the EEG signal was decomposed into several intrinsic mode functions by empirical mode decomposition, then the features were calculated by using the sliding window technique and the autoregressive model, and the classification of the EEG signal was achieved using these features. A seizure detection system was presented in [11], where the system processed the data in stages, including: preprocessing, feature extraction, classifier, and expert system. Overall, the expert system reflects the physician’s experience in seizure detection, and the system looks for spikes in the EEG signal and submits the relevant information to the expert system for the final classification of seizures.

EEG signals contain data of different frequencies and can be transformed from the time domain to the frequency domain by transforming them [12]. The information of different frequencies is analyzed, and features are extracted for the classification of the EEG information. The scheme proposed in [13] uses the discrete wavelet transform to extract EEG features for each frequency sub-band. Then, it relies on the SVM classifier to complete the seizure detection. The scheme has higher sensitivity and specificity in the α and δ bands. In the scheme proposed by [14], the EEG was diagnosed using multiscale principal component analysis; the EEG signal was decomposed using techniques such as wavelet packets, and after relying on statistical methods to extract the features, the seizure detection was performed using machine learning. In [15], the wavelet transform was used to extract wavelet coefficients from the EEG signal, and then, the peaks were obtained from the wavelet coefficients. The peaks were mapped to 3D coordinates, and the Euclidean distance of the 3D coordinates from the origin was characterized by statistical techniques.

Due to the nonstationary nature of EEG, especially during seizures, entropy measures have attracted more attention in the field. The authors of [16] gave two protocols for analyzing the entropy of the EEG, one using a single analysis window, but with each window having different lengths, and the other using multiple windows, each of which can differ in statistical content. In [17], a modified distribution entropy (mDistEn) for epilepsy detection was proposed. mDistEn corresponds to a higher area under the curve (AUC) value compared to fuzzy entropy and distribution entropy and yields a 92% classification accuracy. The scheme in [18] was used for seizure detection, and the scheme was also implemented based on a combination of wavelet analysis and support vector machines. The authors of [19] proposed a hybrid feature-based EEG signal classification scheme to improve the accuracy of seizure detection. The hybrid features contain features commonly used in EEG signals and also the entropy obtained based on the Hilbert–Huang transform proposed by the authors. In [20], the EEG signal was decomposed into nine sub-bands using a tunable-Q wavelet. Then, the entropy, statistical, and fractal features were extracted from the sub-bands, and ensemble learning was used for the EEG classification.

In recent years, deep learning has achieved many successes in the analysis of time series signals, so many studies have tried to use deep learning to process EEG signals [21,22]. In 2017, the authors of [23] implemented seizure detection using a 13-layer deep convolutional neural network. Antoniades et al. [24] performed seizure detection using a four-layer convolutional neural network pair for discrete multichannel intracranial EEG. Different kinds of neural networks have also been used for seizure detection, such as deep belief networks [25], improved SincNet-based networks [26], long short-term memory networks [27], and two-layer long short-term memory networks [28]. In the field of EEG classification, transfer learning also has related results, such as domain-adversarial neural networks [29] and improved EasyTL-based neural networks [30].

In summary, EEG has been widely used for seizure detection, and deep learning has been adopted by numerous studies. However, the neural network structures in these studies are fixed and strongly coupled to the format of the input data. Some of those networks use 1D or 2D convolutional units, and some use 3D convolutional units, as shown in Table 1.

Different studies have used different network models for depth feature extraction and EEG signal classification, and some of these networks were designed based on well-known network models, while some were completely new network models. However, the depth, width, and resolution of these models are difficult to dynamically adjust.

Public datasets used for image classification tests usually contain tens of thousands or even millions of images, which allows neural networks to be adequately trained. If a network can be well trained on these public datasets, it also has the prerequisites for transfer learning, including the use of the trained network for EEG data classification.

## 3. The Proposed Model

The proposed model has five main parts, as shown in Figure 1, which are data decomposition, feature extraction, two-dimensional representation of features, deep feature extraction, and classification. We give the following two phrases their meanings as follows:

1. EEG segment: A data record of the original EEG signal. The time span of this record is determined during the data acquisition;
2. EEG slice: the data slices obtained by decomposing the EEG segment with a sliding time window. In general, a slice contains EEG data with a time span of several seconds.

Part 1 is data decomposition, where the complete EEG data are sliced using a time window. A complete EEG signal is usually an EEG recording over a period of time, and slicing it makes it easier for subsequent processing. Classifying slices usually requires less hardware resources than classifying complete EEG data. In addition, a complete segment of EEG data usually contains both seizure and nonseizure EEG signals, so it is less meaningful to classify the whole EEG signal. Classification of slices, on the other hand, allows for more accurate knowledge of whether a patient is having a seizure at the moment corresponding to the slice.

Decomposing EEG data with a sliding time window, in addition to the length of the time window, affects the slicing results, and whether the time windows overlap each other also affects the results. Compared with no overlap between time windows, the decomposition with an overlap can obtain more slices, which also leads to the local feature information being extracted multiple times.

The length of the time window used in different studies is not necessarily the same, which also correlates with the feature extraction method used. Part 2 is the extraction of features, the main purpose of which is to reduce the dimensionality of the slices while retaining the information used for EEG classification. The features being extracted are generally different when faced with different problems. In the field of EEG-based seizure detection, frequency and time–frequency domain features are often extracted, so the model in this paper mainly extracts frequency and time–frequency domain features.

The model extracts time–frequency domain features with the wavelet transform for slices generated from single-channel EEG data and uses them for representation. For slices generated from multichannel EEG data, the fast Fourier transform is used to extract the frequency domain features of each channel, and then, the features obtained from different channels are unified in Part 3.

The representation of features in Part 3 consists of two steps, first characterizing the features as pictures, called the 2D representation of the features. Although features can be characterized in one or more dimensions, there are many advantages to using 2D representation:

1. When representing the slices generated from single-channel EEG signals, temporal information can be included in the images generated by the representation. When representing slices generated from multichannel EEG signals, the picture contains the information of each channel. In contrast, the one-dimensional representation contains less information, while the multidimensional representation contains more information, but often requires more computational resources;
2. Most neural networks use convolutional layers, and the amount of computation required to perform convolutional operations increases rapidly as the dimensionality of the input data increases. Because of the black-box property of neural networks, it is more difficult for them to perform dimensionality reduction on the input data. The use of images as input data also considers the need for computational resources;
3. Many excellent neural networks, which are tested based on publicly available image datasets, give these networks the ability to perform transfer learning. Through transfer learning, the neural network trained with the public dataset can be used as the base network, and the base network can be further trained using the represented images to obtain the neural network weights for EEG signal classification.

**Figure 2:**
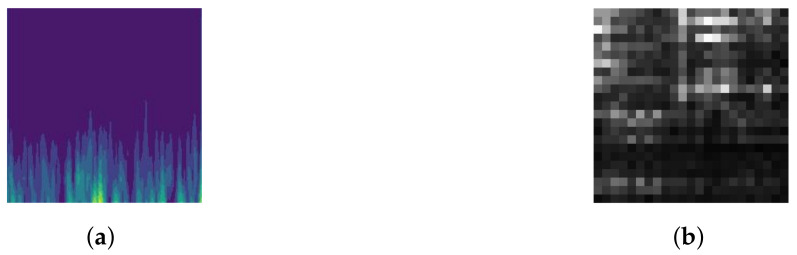
Representation of the features. (**a**) An example (Bonn dataset): the result of a 2D representation. (**b**) An example (CHB-MIT dataset): the result of a 2D representation.

For the slices generated by single-channel EEG, the results obtained from the wavelet transform can be characterized in two dimensions, with time and frequency as the horizontal and vertical coordinates, respectively. The contour map drawn with the result is the representation of the slice’s features, as shown in Figure 2a.

For the slices generated by multichannel EEG, the FFT is performed on the data of each channel. With the frequency as the horizontal coordinate and the channel as the vertical coordinate, the result of the FFT is the value in the coordinate. Take a 23-channel slice as an example, with a 1 Hz sampling interval and a frequency band of 1–23 Hz. With frequency as the horizontal coordinate and 23 channels as the vertical coordinate, a 23×23-pixel picture is obtained, which is the 2D representation of the slice, as shown in Figure 2b. If the frequency band is extended to 1–46 Hz, then the representation of the features is a 23×46-pixel picture.

After the 2D representation of the slice’s features to obtain the images, the second step in Part 3 is the image enhancement, which is an optional step. This step performs operations such as rotating or flipping the images, and it aims at increasing the training samples or making the trained neural network more robust.

Part 4 extracts the depth features of the image. The image is scaled to the size needed by the neural network and then normalized, and the result is used as the input data for the network. Normalization usually allows the neural network to converge faster during training and prevents the gradient from disappearing.

The trained network can extract depth features from the input images, which in general are the second layer of the inverse order in the neural net. The classifier takes the depth features as the input and gives the classification result. In Part 4, EfficientNet is used to extract the depth features, and such a scalable neural network has many advantages, as follows:

1. The network can be changed in terms of depth, width, and resolution when facing different hardware resource-constrained deployment environments. This allows the model to have a stable performance under different hardware resource constraints;
2. It combines the characteristics of different input data, increases the depth, width, and resolution of the network to extract more information from the input data, and improves the performance of the proposed model;
3. The difficulty of neural network scaling includes determining the scaling ratio among the three factors of depth, width, and resolution, which, if balanced well, can obtain better classification accuracy than if one factor is expanded individually. Balancing the scaling ratio of the three is difficult, and EfficientNet provides an available network scaling scheme.

**Figure 3:**
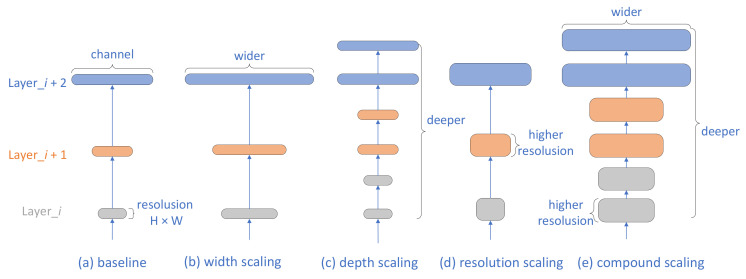
Neural network scaling.

The scalability of a neural network refers to the scalability on three factors: depth, width, and resolution. Taking the baseline network shown in Figure 3a as an example [2], the scaling of the width, which refers to the change in the number of channels, is shown in Figure 3b. Increasing the width of the network alone will obtain more features, but it is often difficult to learn deeper features because of the limitation of the network depth. The change in depth refers to the change in the number of layers, as shown in Figure 3c. Expanding the depth of the network alone can obtain more complex features, but excessively increasing the depth without changing the other dimensions tends to cause the gradient to disappear, which makes the training of the network difficult. The size of the resolution is related to the input data, in other words, as shown in Figure 3d. Increasing the resolution of the input image alone can result in a greater resolution, but this increases the amount of operations. Not increasing the depth and width of the network can easily lead to a decrease in the gain of increasing the resolution. To better improve the network classification accuracy, scaling in EfficientNet is performed on all three factors simultaneously, as shown in Figure 3e.

The baseline network of EfficientNet is called EfficientNet-B0, and then, different scaling ratios generate EfficientNet-B1, B2, B3, B4, B5, B6, and B7. The structure of EfficientNet-B0 is shown in Table 2; while B1-B7 require different resolutions of input data, as shown in Table 3, when the resolution of the input data is different from the network required resolution, it is necessary to use the first step in Part 4 to convert the input data to the required resolution.

In Part 5, the classification of depth features is performed by the classifiers, which include softmax, KNN, SVM, etc. The softmax classifier is used in EfficientNet. The classification results of the depth features are obtained, which can also be used as the classification results of the slices to determine whether a seizure is detected or not.

## 4. Experiments

In this section, we first present two popular datasets that are commonly used in seizure studies. Then, the details of the experiments are presented, and finally, the models are compared with some popular methods for classification accuracy. Because of the limitation of the hardware resources of the experimental platform, only EfficientNet-B0, B1, B2, B3, and B4 were tested in the experiments.

### 4.1. Dataset and Performance Indices

The Bonn database was published in 2001 [49]. Bonn contains five subsets, Set A, Set B, Set C, Set D, and Set E. Each subset contains 100 single-channel EEG clips of 23.6 s in duration, which were manually selected from long-range multichannel EEGs and removed from interference such as muscle and eye movement artifacts. Set A and Set B are scalp EEGs from 5 healthy individuals with eyes open and closed, the international 10–20 system, sampled at 173.61 Hz. Set C, Set D, and Set E are intracranial EEGs from five patients with epilepsy whose lesions were in the hippocampal structures. Their seizures were controlled after partial removal of the hippocampal structures. The electrodes of Set D were located at the lesion, and the electrodes of Set C were located on the opposite side of the lesion. Set E included electrodes of Set C and Set D, in addition to some electrodes located in the lateral and basal regions of the neocortex. Set C and Set D were taken from the interictal period, and Set E was taken from the seizure period, both with a sampling frequency of 173.61 Hz.

Another dataset is the CHB-MIT [50,51] provided by Boston Children’s Hospital, and this dataset has also been widely used in studies of seizure detection. The dataset contains 24 sets of scalp EEG data, as shown in Section 4.2. These data were acquired from 23 patients; chb01 and chb21 were acquired from the same patient, with a time interval of 1.5 years between acquisitions. The international 10–20 system acquires signals at 256 Hz, 16 bit. Each set has 9–42 consecutive multichannel EEG clips, some of which recorded seizures. The duration of the EEG clips was mostly 1 h, with a small number of clips of 2–4 h, and some clips were relatively short because the acquisition process was artificially interfered with. In order to evaluate the effect of the surgical intervention, no antiseizure drugs were used during the data collection.

Three common evaluation indices were used to analyze the results of the model experiments, namely accuracy, sensitivity, and specificity, which are defined as follows:

(1) $$\mathrm{Accuracy}=\frac{TP+TN}{TP+TN+FP+FN}\times 100\%$$

(2) $$\mathrm{Sensitivity}=\frac{TP}{TP+FN}\times 100\%$$

(3) $$\mathrm{Specificity}=\frac{TN}{TN+FP}\times 100\%$$

The operation of classifying an input image is called a case in this paper. *TP*, *FP*, *TN*, and *FN* are defined as follows: *TP*: the number of cases where the predictions are seizure state and correct; *FP*: the number of cases where the predictions are seizure state and incorrect; *TN*: the number of cases where the predictions are normal state and correct; *FN*: the number of cases where the predictions are normal state and incorrect.

Accuracy is the proportion of correctly classified seizure and nonseizure images. Sensitivity is the proportion of correctly classified seizure images. Specificity is the proportion of correctly classified nonseizure images.

### 4.2. Model Performance

**Figure 4:**
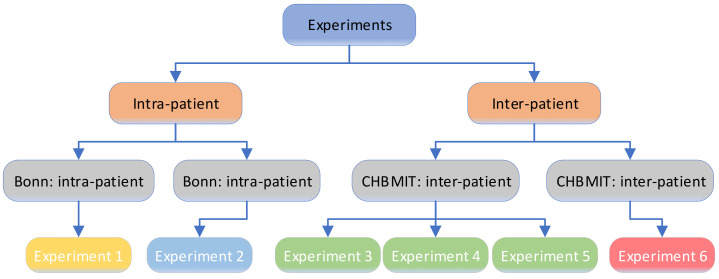
Experiments for model performance testing.

The experiments were divided into intrapatient and interpatient mode and were carried out on the Bonn and CHB-MIT datasets, as shown in Figure 4. The experiments consisted of four types of tests, with a total of six experiments. The proportion of seizure versus nonseizure was 1:1, and the size of the time window changed in different experiments. In intrapatient mode, the seizure data and nonseizure data of all patients were pooled, and a portion of the data were randomly selected for training the neural network, while the rest were used as the test dataset; the experiment was repeated to find the average value. In interpatient mode, the data of some patients were used to train the neural network, and the data of other patients were used as the test set; the experiment is repeated was find the average value.

Bonn: intrapatient mode. Set D was used as the data during seizures, and Set E was used as the data during nonseizures. Data from all 5 patients were pooled and randomly assigned to the training set, validation set, and test set.

Objective of Experiment 1: To observe the model classification performance when changing the length of the time windows.

The main parameter configuration: The model uses EfficientNet-B0 as the neural network and 50% overlap between time windows. The time window had eight different values: 1, 2, 3, 4, 5, 10, 15, 23.6 s. The resolution of the image obtained by feature representation was 224 × 224 pixels; the fundamental wave was cgau8; and the total scale was 10.

The experiment results: The experiment results are shown in Table 4, showing the statistics of the classification results of the model at different time window lengths.

Bonn: interpatient mode. Set D was used as the data during seizures and Set E as the data during nonseizures. The data of one patient were used as the test set, and the data of the other four patients were used as the training and validation sets.

Objective of Experiment 2: To observe the model classification performance when scaling the EfficientNet network and the impact of using transfer learning or not.

The main parameter configuration: Considering the results of Experiment 1, the time window was chosen to be 3 s; the image resolution was consistent with the requirements of the neural network; the fundamental wave was cgau8; and the total scale was 10. Table 5 shows the classification results of the model with different network scalings when transfer learning was used.

The experiment results: Table 6 shows the classification results when transfer learning was not used.

**Table 5:** The results of Experiment 2 when transfer learning was used. Acc, Sen and Spe are the abbreviations for accuracy, sensitivity, and specificity, respectively.

Network	Patient 1			Patient 2			Patient 3			Patient 4			Patient 5		
	Acc (%)	Sen (%)	Spe (%)	Acc (%)	Sen (%)	Spe (%)	Acc (%)	Sen (%)	Spe (%)	Acc (%)	Sen (%)	Spe (%)	Acc (%)	Sen (%)	Spe (%)
B0	95.00	96.43	93.57	97.50	97.86	97.14	93.93	92.86	95.00	95.00	95.71	94.29	95.36	92.14	98.57
B1	95.71	95.71	95.71	96.79	97.86	95.71	94.64	93.57	95.71	95.36	96.43	94.29	96.07	96.43	95.71
B2	96.43	96.43	96.43	97.86	99.29	96.43	93.57	95.71	91.43	96.43	95.71	97.14	95.71	96.43	95.00
B3	96.79	95.71	97.86	96.07	98.57	93.57	94.29	93.57	95.00	94.29	95.00	93.57	96.79	97.14	96.43
B4	95.71	95.00	96.43	94.29	99.65	96.97	93.93	92.14	95.71	95.71	97.14	94.29	95.36	97.14	93.57

CHB-MIT: intrapatient mode. The information of the patients is shown in Table 7. Male (M) patients whose age interval was no less than 5 years with respect to other male patients were selected, and there were four patients: chb02, chb04, chb10, and chb15. Because chb08 and chb10 were close in age, only chb10 was selected. We constructed four groups as shown in Table 8. In each group, there were EEG data from one M and one female (F), and the two patients were as close in age as possible. Equal-duration seizure period and nonseizure period data were used for the experiment.

Objective of Experiment 3: The performance of the model classification was observed by varying the length of the time window and selecting different frequency bands.

The main parameter configuration: The data from all patients in the 4 groups were pooled and randomly assigned to the training set, validation set, and test set. The time window had five different values: 1, 2, 3, 4, 5 s. The model used EfficientNet-B0 as the neural network and 50% overlap between time windows. Three frequency bands were selected, 1–23, 12–34, and 23–45 Hz. The original resolution of the images was 23 × 23 pixels, which was reshaped to the resolution needed by the network.

The experiment results: The classification results of the model with different time window lengths and different frequency bands were counted, and the results are shown in Table 9.

Objective of Experiment 4: The performance of the model classification was observed by varying the length of the time window.

The main parameter configuration: The data from all patients in the 4 groups were pooled and randomly assigned to the training set, validation set, and test set. The time window had five different values: 1, 2, 3, 4, 5 s. The model used EfficientNet-B0 as the neural network and 50% overlap between time windows, in addition to a frequency band of 1–46 Hz. The original resolution of the image was 23 × 46 pixels, which was reshaped to the resolution needed by the network.

The experiment results: The classification performance of the model at different time window lengths was statistically measured, and the experimental results are shown in Table 10.

Objective of Experiment 5: The data from a single group were used as all experimental data to observe the classification results in the intrapatient model.

The main parameter configuration: In Experiments 3 and 4, the data from Groups 1–4 were aggregated to form the entire experimental data, while in Experiment 5, classification experiments were conducted using the data from each group separately. In other words, the data of each group were divided into three parts: training set, validation set, and test set. Overall, the accuracy in Experiment 4 was higher than for the data in Experiment 3, so the frequency band of 1–46 was used in Experiment 5. The time window was 1 s in this experiment, and the reason was that the two largest values of the accuracy in Experiment 4 occurred at the time window of 1 s and 3 s, with the former being 97.06 and the latter being 97.77. More images were obtained with a time window length of 1 s than 3 s, so a time window of 1 s with 50% overlap was set in Experiment 5.

The experiment results: The model used EfficientNet-B0 as the neural network, and the classification results are shown in Table 11.

CHB-MIT: interpatient mode. Chb6, chb12, chb21, and chb24 were excluded from the CHB-MIT dataset, and the remainder constituted the full data of the experiment. chb6 and chb12 were excluded because these two patients were no older than 2 y, and it is generally believed that EEG data for seizures in young infants are different from those in adults, so seizures in infants are best studied separately from those in adults. chb01 and chb21 were collected from the same patient, and only chb01 was retained. chb24 was excluded because the patient information was unclear.

Objective of Experiment 6: When using transfer learning, the model classification performance was observed when the EfficientNet network was scaled.

The main parameter configuration: A group was used as the test set, and the entire experimental data excluding this group was used as the training and validation set. The length of the time window was 3 s, with 50% overlap between time windows and a frequency band of 1–46 Hz.

The experiment results: The original resolution of the images was 23 × 46 pixels, reshaped to the resolution needed by the network, and the experimental results are shown in Table 12.

### 4.3. Comparison with Other Schemes

Many of the schemes for seizure detection only provide results in intrapatient mode, and a comparison of the proposed model with these schemes is shown in Table 13 and Table 14, Table 13 is based on Bonn and Table 14 on CHB-MIT. It should be noted that the methods of EEG decomposition and feature representation were not necessarily the same in different schemes, so these experiments, although based on the same dataset, did not necessarily have the same input data for the neural network.

## 5. Discussion

**Figure 5:**
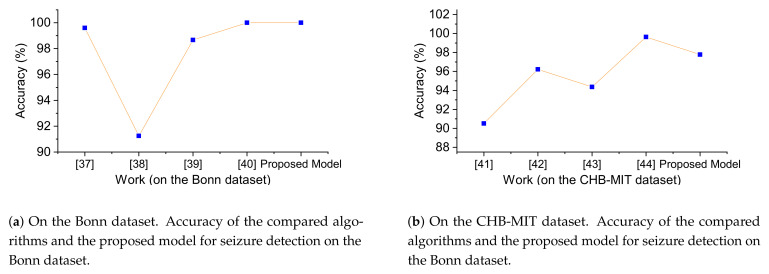
Algorithm comparison. The accuracy of the compared algorithms and the proposed model for seizure detection on the two datasets. The *x*-axis is for each algorithm and the *y*-axis for accuracy.

The model was tested on the Bonn dataset and the CHB-MIT dataset, and the classification accuracy was compared with some popular neural networks employing two-dimensional convolution. When using the Bonn dataset, the best intragroup classification accuracy of the proposed scheme reached 100, which was consistent with the best result in the comparison algorithm, as shown in Figure 5a. When using the CHB-MIT dataset, the proposed solution did not have the highest classification accuracy, but it also achieved a good value, as shown in Figure 5b. Overall, most of the seizure detection algorithms were tested based on a single dataset, while the proposed model was tested on two datasets and performed well, which also indicated the effectiveness and cross-dataset generality of the proposed scheme.

**Figure 6:**
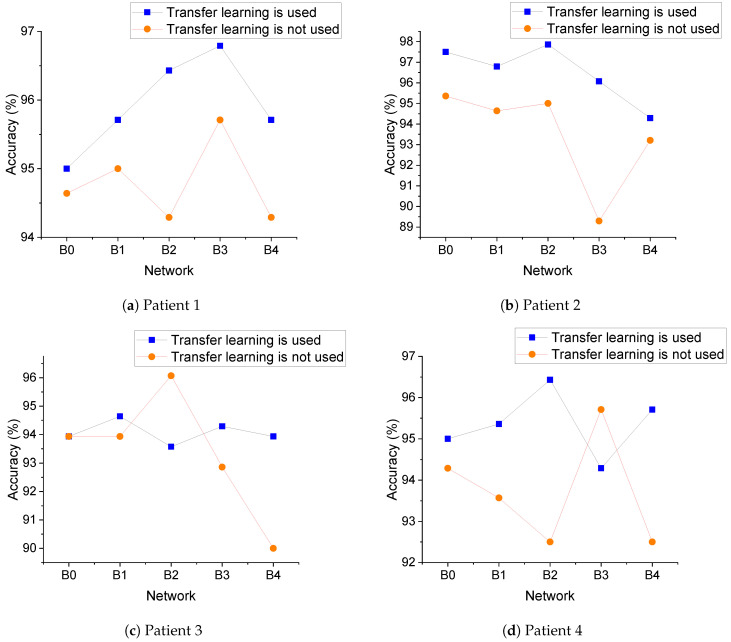

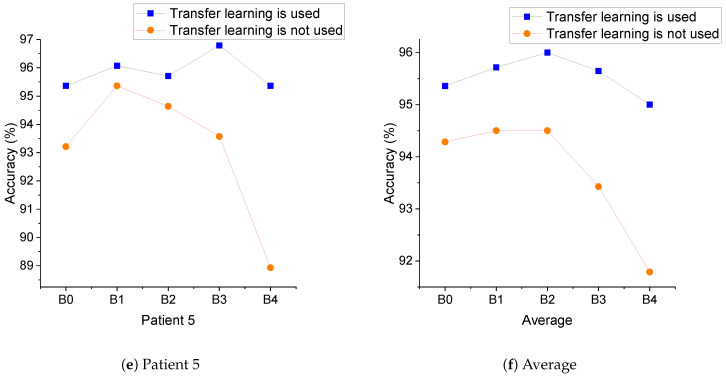
Transfer learning. The EfficientNet trained on the ImageNet dataset. The network was then trained using the seizure dataset to obtain the final network weights.

Here, we analyze the effect of transfer learning on the model performance. In Experiment 2-1, the model used transfer learning, based on a network trained by EfficientNet on the ImageNet dataset, and then trained the network using the seizure dataset to obtain the final network weights. In Experiment 2-2, no transfer learning was used, and only the seizure dataset was used to train the network to obtain the final network weights. The data used in both experiments were the same, and the experiments yielded 25 accuracy values. Twenty-two accuracy values in Experiment 2-1 were greater than the values in Experiment 2-1, and one accuracy value was the same. The accuracy of using EfficientNet-B0, B1, B2, B3, and B4 for each patient is shown in Figure 6a–e, and the average accuracy of each network for five patients is shown in Figure 6f. Overall, transfer learning improved the accuracy and allowed the model to obtain better performance.

**Figure 7:**
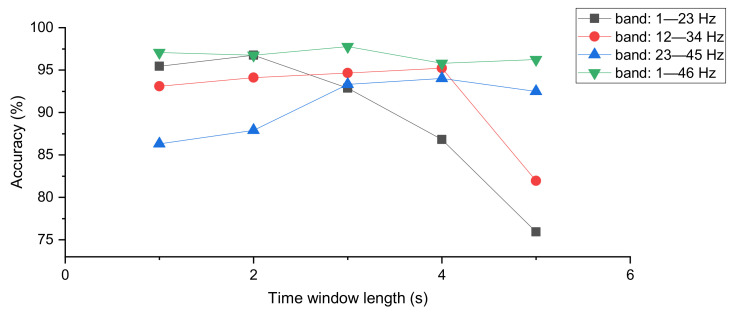
The selection of the frequency bands. Accuracy obtained by combining different frequency bands with different time window lengths.

The EEG signal can be regarded as a superposition of different frequency signals, and here, we analyze the effect of choosing different frequency bands on the classification results. In Experiment 3, the width of the frequency band was set to 23 Hz, and the two largest accuracy values appeared in the frequency band of 1–23 Hz. It is also noteworthy that the classification accuracy of all three bands had a tendency to increase and then decrease when the time window was gradually increased, as shown in Figure 7. Therefore, the frequency bands needed to be considered simultaneously when determining the length of the time window. In Experiment 4, the overall result of the accuracy was higher than that of Experiment 3, which shows that using wider frequency bands can effectively improve the performance of the accuracy.

**Figure 8:**
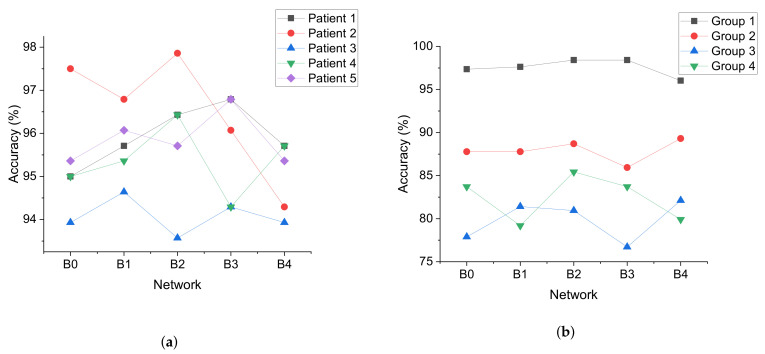
Accuracy when scaling the network. Scaling of the depth, width, and resolution of the network. (**a**) Data from Table 5, the result of Experiment 2 when transfer learning was used. (**b**) Data from Table 12, the result of Experiment 6.

The effect of network scaling on model performance was obvious. In Experiment 2 and Experiment 6, the maximum value of the accuracy for each patient or group was obtained by different networks, as shown in Figure 8. The maximum value of the accuracy obtained by EfficientNet-B0 was 1, by EfficientNet-B1 2, by EfficientNet-B2 5, and by EfficientNet-B3 5, and the maximum value obtained by EfficientNet-B4 was 2. It can be seen that the maximum values of the accuracy were mainly obtained by EfficientNet-B2 and EfficientNet-B3. This shows that the network scaling can improve the performance of the model compared with EfficientNet-B0, while the maximum values obtained by EfficientNet-B4 were not the most, probably because there were not enough training data and the network was not sufficiently trained.

Based on the Bonn dataset, the results of the intrapatient and interpatient mode were obtained from Experiment 1 and Experiment 2. When the time window was set to 3 s, the accuracy in Experiment 1 was 97.14 and the maximum accuracy for the five patients in Experiment 2 was 96.79, 97.86, 94.64, 96.43, and 96.79 with a mean value of 96.502. The accuracy of the interpatient mode was lower than the accuracy of the interpatient mode.

Based on the CHB-MIT dataset, the results of the intrapatient and interpatient mode were obtained by Experiment 5 and Experiment 6. The mean accuracy of all groups in Experiment 5 was 97.575, and the maximum accuracy of each patient in Experiment 6 was 98.41, 89.30, 82.12, and 85.43 with a mean value of 88.815. The accuracy of the interpatient model was lower than the accuracy of the intrapatient model.

**Figure 9:**
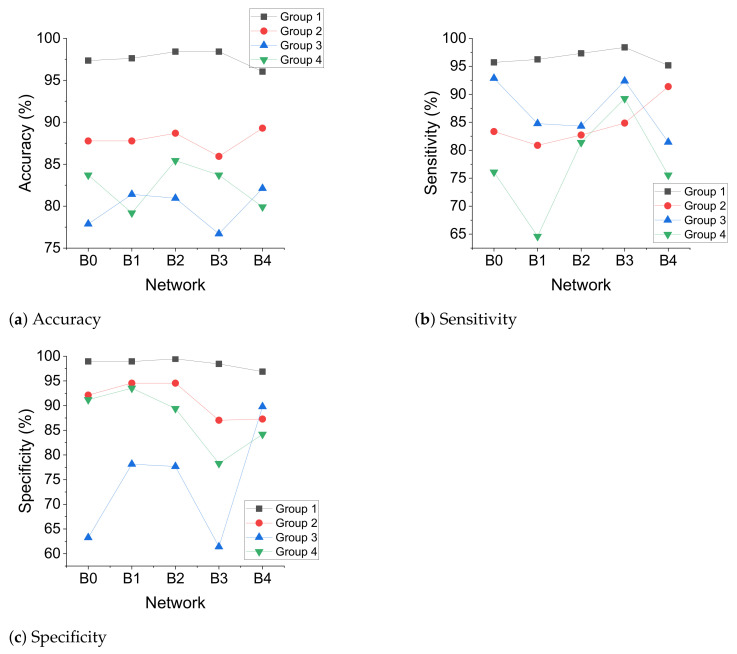
When the network is scaled, comparing the classification performance of Group 3 and other groups in the interpatient mode.

In Experiment 6, the maximum value of the accuracy for Group 3 was 82.12, which was smaller than the other groups, as shown in Figure 9a. This may be because the patients in Group 3 were all three years old and very young children have different EEG characteristics during seizures than adults. Group 3 performed better on the sensitivity and worse on the specificity compared to the other groups, as shown in Figure 9b,c. This suggests that the reliability of the data from adult seizures to predict infant seizures was stronger than using data from adult nonseizures to predict nonseizures in infants.

The advantages of the proposed model: The proposed model consists of five parts that are weakly coupled to each other. For example, when the model determines the resolution of the 2D representation image, if the feature extraction algorithm is replaced, there is no need to modify the other parts as long as the image with the same resolution is finally generated. The weak coupling of the parts allows the different parts of the proposed model to be quickly upgraded or even quickly replaced with new algorithms. The disadvantages of the proposed model: The parameter settings for different parts of the model rely on experience, which makes the classification performance unstable when the model is targeted at different datasets and classification purposes. In addition, feature extraction is currently limited to the time domain and the time–frequency domain, which reduces the number of ways to extract different features for better classification performance, and further research is needed on how to extract more kinds of features.

## 6. Conclusions and Future Work

In this study, a classification model based on 2D representation of EEGs and a scalable neural network was proposed to improve the adaptability of the seizure detection model to different hardware resource constraints and to improve the convenience of adopting new algorithms for different parts in the model. The model was tested on two different seizure datasets, and the results showed the generality and good classification accuracy of the model in seizure detection. Although the proposed model is effective at seizure detection, it still has some shortcomings. The model can be used for single-channel and multichannel EEG, but it is difficult to classify EEG signals with dynamic changes in the number of channels. Although the problem can be transformed into the classification of multiple single-channel EEG signals, the final classification results can be obtained with techniques such as fuzzy systems, but the model needs more work to deal with these problems, which is one of our future research directions.

## Figures and Tables

**Table 1:** Neural networks with different convolutional dimensions used.

Work	Dataset	Convolutional Dimension	Accuracy (%)
[31]	TUH	1D network	79.34
[32]	Clinical	1D network	89.73
[33]	CHB-MIT	1D network	84
[34]	Bonn	1D network	97.27
[35]	Clinical	1D network	83.86
[36]	Bonn	1D network	86.67
[37]	Bonn	2D network	99.60
[38]	Bonn	2D network	91.25
[39]	Bonn	2D network	98.67
[40]	Bonn	2D network	100
[41]	CHB-MIT	2D network	90.50
[42]	CHB-MIT	2D network	96.22
[43]	CHB-MIT	2D network	94.37
[44]	CHB-MIT	2D network	99.63
[45]	Clinical	2D network	95.19
[46]	Bern Barcelona	2D network	91.8
[47]	UCI	2d network	85.3
[48]	Clinical	3D network	99.4

**Table 2:** Baseline network: EfficientNet-B0.

Stage *i*	Operator	Resolution	Channel	Layers
1	Conv3×3	224 × 224	32	1
2	MBConv1, k3×3	112 × 112	16	1
3	MBConv6, k3×3	112 × 112	24	2
4	MBConv6, k5×5	56 × 56	40	2
5	MBConv6, k3×3	28 × 28	80	3
6	MBConv6, k5×5	14 × 14	112	3
7	MBConv6, k5×5	14 × 14	192	4
8	MBConv6, k3×3	7 × 7	320	1
9	Conv1×1 and pooling and FC	7 × 7	1280	1

**Table 3:** EfficientNet compound scaling settings.

Network	Input_Resolution	Width_Coefficient	Depth_Coefficient
EfficientNet-B0	224 × 224	1.0	1.0
EfficientNet-B1	240 × 240	1.0	1.1
EfficientNet-B2	260 × 260	1.1	1.2
EfficientNet-B3	300 × 300	1.2	1.4
EfficientNet-B4	380 × 380	1.4	1.8
EfficientNet-B5	456 × 456	1.6	2.2
EfficientNet-B6	528 × 528	1.8	2.6
EfficientNet-B7	600 × 600	2.0	3.1

**Table 4:** The results of Experiment 1.

Time Window Length (s)	Accuracy (%)	Sensitivity (%)	Specificity (%)
1	94.13	93.04	95.22
2	95.91	92.73	99.09
3	97.14	98.57	95.71
4	95.00	93.33	96.67
5	98.75	100	97.50
10	97.50	95.00	100
15	96.67	93.33	100
23.6	100	100	100

**Table 6:** The results of Experiment 2 when transfer learning was not used. Acc, Sen and Spe are the abbreviations for accuracy, sensitivity, and specificity, respectively.

Network	Patient 1			Patient 2			Patient 3			Patient 4			Patient 5		
	Acc (%)	Sen (%)	Spe (%)	Acc (%)	Sen (%)	Spe (%)	Acc (%)	Sen (%)	Spe (%)	Acc (%)	Sen (%)	Spe (%)	Acc (%)	Sen (%)	Spe (%)
B0	94.64	95.71	93.57	95.36	96.43	94.29	93.93	95.71	92.14	94.29	97.14	91.43	93.21	91.43	95.00
B1	95.00	96.43	93.57	94.64	97.86	91.43	93.93	92.86	95.00	93.57	96.43	90.71	95.36	96.43	94.29
B2	94.29	96.43	92.14	95.00	97.86	92.14	96.07	94.29	97.86	92.50	95.71	89.29	94.64	98.57	90.71
B3	95.71	96.43	95.00	89.29	90.00	88.57	92.86	94.29	91.43	95.71	96.43	95.00	93.57	91.43	95.71
B4	94.29	95.71	92.86	93.21	96.43	90.00	90.00	92.86	87.14	92.50	97.86	87.14	88.93	91.43	86.43

**Table 7:** Gender and age of the data utilized.

Patient Index	Gender	Age	Patient Index	Gender	Age
chb01	F	11	chb13	F	3
chb02	M	11	chb14	F	9
chb03	F	14	chb15	M	16
chb04	M	22	chb16	F	7
chb05	F	7	chb17	F	12
chb06	F	1.5	chb18	F	18
chb07	F	14.5	chb19	F	19
chb08	M	3.5	chb20	F	6
chb09	F	10	chb21	F	13
chb10	M	3	chb22	F	9
chb11	F	12	chb23	F	6
chb12	F	2	chb24	/	/

**Table 8:** Eight patients were selected from CHB-MIT and divided into four groups.

Group Index	Patient Index	Gender	Age
Group 1	chb02	M	11
	chb01	F	11
Group 2	chb04	M	22
	chb19	F	19
Group 3	chb10	M	3
	chb13	F	3
Group 4	chb15	M	16
	chb07	F	14.5

**Table 9:** The results of Experiment 3. Acc, Sen and Spe are the abbreviations for accuracy, sensitivity, and specificity, respectively.

Time Window Length (s)	Frequency Band: 1–23 Hz			Frequency Band: 12–34 Hz			Frequency Band: 23–45 Hz		
	Acc (%)	Sen (%)	Spe (%)	Acc (%)	Sen (%)	Spe (%)	Acc (%)	Sen (%)	Spe (%)
1	95.44	92.58	98.25	93.09	90.80	95.34	86.32	83.38	89.21
2	96.76	95.83	97.66	94.10	91.07	97.08	87.91	91.07	84.80
3	92.86	95.58	90.09	94.64	93.69	95.58	93.30	90.99	95.58
4	86.83	78.31	95.24	95.21	92.77	97.62	94.01	92.77	95.24
5	75.94	71.21	80.60	81.95	74.24	89.55	92.48	90.91	94.03

**Table 10:** The results of Experiment 4.

Time Window Length (s)	Accuracy (%)	Sensitivity (%)	Specificity (%)
1	97.06	97.03	97.08
2	96.76	95.24	98.25
3	97.77	97.30	98.23
4	95.81	93.98	97.62
5	96.24	96.97	95.52

**Table 11:** The results of Experiment 5.

Group Index	Accuracy (%)	Sensitivity (%)	Specificity (%)
Group 1	100	100	100
Group 2	98.02	96	100
Group 3	94.12	88.89	98.63
Group 4	98.16	97.88	98.44

**Table 12:** The results of Experiment 6. Acc, Sen and Spe are the abbreviations for accuracy, sensitivity, and specificity, respectively.

Network	Test Group: Group 1			Test Group: Group 2			Test Group: Group 3			Test Group: Group 4		
	Acc (%)	Sen (%)	Spe (%)	Acc (%)	Sen (%)	Spe (%)	Acc (%)	Sen (%)	Spe (%)	Acc (%)	Sen (%)	Spe (%)
B0	97.35	95.70	98.96	87.77	83.33	92.12	77.88	92.86	63.26	83.71	76.09	91.20
B1	97.62	96.24	98.96	87.77	80.86	94.55	81.41	84.76	78.14	79.19	64.60	93.54
B2	98.41	97.31	99.48	88.69	82.72	94.55	80.94	84.29	77.67	85.43	81.39	89.41
B3	98.41	98.39	98.44	85.93	84.85	87.04	76.71	92.38	61.40	83.71	89.23	78.28
B4	96.03	95.16	96.88	89.30	91.36	87.27	82.12	81.43	89.79	79.91	75.55	84.20

**Table 13:** Accuracy of some seizure detection methods on the Bonn dataset.

Work	Dataset	Tools	Convolutional Dimension	Accuracy (%)
[37]	Bonn	MATLAB	2D network	99.60
[38]	Bonn	Keras	2D network	91.25
[39]	Bonn	MATLAB	2D network	98.67
[40]	Bonn	Keras	2D network	100
Proposed Model	Bonn	PyTorch	2D network	100

**Table 14:** Accuracy of some seizure detection methods on the CHB-MIT dataset.

Work	Dataset	Tools	Convolutional Dimension	Accuracy (%)
[41]	CHB-MIT	NA	2D network	90.50
[42]	CHB-MIT	PyTorch	2D network	96.22
[43]	CHB-MIT	PyTorch	2D network	94.37
[44]	CHB-MIT	PyTorch	2D network	99.63
Proposed Model	CHB-MIT	PyTorch	2D network	97.77

## Data Availability

All data used during the study appear in the submitted article.
